# Circulating N-Terminal Probrain Natriuretic Peptide Levels in Relation to Ischemic Stroke and Its Subtypes: A Mendelian Randomization Study

**DOI:** 10.3389/fgene.2022.795479

**Published:** 2022-02-22

**Authors:** Ming Li, Yi Xu, Jiaqi Wu, Chuanjie Wu, Ang Li, Xunming Ji

**Affiliations:** ^1^ China-America Institute of Neurology, Xuanwu Hospital, Capital Medical University, Beijing, China; ^2^ Department of Neuroscience, Xuanwu Hospital, Capital Medical University, Beijing, China; ^3^ Beijing Advanced Innovation Center for Big Data-Based Precision Medicine, School of Biological Science and Medical Engineering, Beihang University, Beijing, China; ^4^ Department of Biomedical Engineering, Columbia University, New York City, NY, United States

**Keywords:** mendelian randomization, single nucleotide polymorphisms, N-terminal pro-brain natriuretic peptide, stroke, risk predictor

## Abstract

Mendelian randomization was used to evaluate the potential causal association between N-terminal probrain natriuretic peptide (NT-proBNP) and ischemic stroke based on summary statistics data from large-scale genome-wide association studies. Three single-nucleotide polymorphisms (SNPs) rs198389, rs13107325, and rs11105306 associated with NT-proBNP levels found in large general populations and in patients with acute heart disease were used as instrumental variables. The results of genetic association analysis of each single SNP show that there is no significant association between NT-proBNP levels and ischemic stroke or its subtypes, whereas rs198389 alone has a suggestive association with large-artery atherosclerosis stroke. The MR analysis of three SNPs shows that NT-proBNP levels may reduce the risk of small-vessel occlusion stroke suggestively. This genetic analysis provides insights into the pathophysiology and treatment of ischemic stroke.

## Introduction

Stroke is the second major cause of global death with a mortality rate of approximately 5.5 million/year and poses a huge financial burden to family members and public health (Donkor, 2018). The study of INTERSTROKE presents 10 potentially modifiable risk factors that are associated with around 90% of acute strokes (O'Donnell et al., 2016), and according to the data from the INTERHEART study, those factors also account for the great majority of the risk of myocardial infarction (Yusuf et al., 2004). Therefore, it is generally acknowledged that a bidirectional interaction exists between brain damage and heart dysfunction (Chen et al., 2017; Scheitz et al., 2018), which may share overlapping cell death pathways (Gonzales-Portillo et al., 2016).

N-terminal probrain natriuretic peptide (NT-proBNP) is an N-terminal fragment of brain natriuretic peptide (BNP), released from the heart muscle in response to the blood pressure and volume overload (Daniels and Maisel, 2007). This factor is widely used in the clinic as a prognostic biomarker to predict mortality in patients with coronary artery disease (CAD), atrial fibrillation, and heart failure (Johansson et al., 2016). Compared with BNP, NT-proBNP presents a longer circulating half-life, higher plasma concentration, and greater diagnostic sensitivity. Due to the connections between cardiac dysfunction and stroke, NT-proBNP is supposed to be a potential predictor for the risk of ischemic stroke (Zhao et al., 2020a).

The relationship between NT-proBNP and risks of stroke remains a popular research subject. The related research dates back to 1996 (Rubattu et al., 1996). The scientific community has increasing interest in this area from 2010 (García-Berrocoso et al., 2010; Kim et al., 2010; Quan et al., 2010) to 2020 (Bhatia et al., 2020; Harpaz et al., 2020; Hotsuki et al., 2020; Khan and Kamal, 2020; Medranda et al., 2020; Rubattu et al., 2020; Shirotani et al., 2020; Tonomura et al., 2020; Wang et al., 2020; Watson et al., 2020; Yang et al., 2020; Zhao et al., 2020b). Several studies explore and identify variable degrees of correlation in different types of stroke. The data from the population-based Atherosclerosis Risk in Communities (ARIC) study shows that NT-proBNP was associated positively with total stroke, non-lacunar ischemic, as well as cardioembolic stroke, but not with lacunar or hemorrhagic stroke (Folsom et al., 2013). NT-proBNP is a strong predictor of atrial fibrillation, which makes it a contributor to the incidence of cardioembolic stroke (Yang et al., 2014). A recent study indicates that serum levels of NT-proBNP higher than 800 pg/ml obtained within 72 h after a transient ischemic attack were associated with an increased risk of stroke (Rodríguez-Castro et al., 2020). More interestingly, in 2019, based on the Biomarkers for Cardiovascular Risk Assessment in Europe-Consortium, Castelnuovo et al. (2019) analyzed data of 58,173 participants free of stroke from six community-based cohort studies and found that, in the European group, levels of NT-proBNP have positive association with risk of ischemic and hemorrhagic stroke, independent from several other conditions and risk factors. These findings cannot be easily explained by the known physiological function of BNP.

The role of NT-proBNP in the incidence of stroke became an unsolved question. A meta-analysis of 16 studies suggests that NT-proBNP provides minor clinical predictive values for the prediction of stroke mortality (García-Berrocoso et al., 2013). According to the research of George et al. (Giannakoulas et al., 2005), no significant correlation was observed between NT-proBNP levels and stroke severity or infarct volume. Another study also denied this association in terms of functional outcomes (Etgen et al., 2005). Evidence suggests the causal relationships of natriuretic peptides to endothelial permeability, which might predispose people to atherosclerosis and hemorrhages (Lee et al., 2007; Lin et al., 2007; Kuhn, 2012; Cannone et al., 2013). Therefore, some researchers hypothesized that NT-proBNP may be involved in the causal physiological path for stroke incidence or be a causal risk factor of stroke (Cushman et al., 2014; Di Castelnuovo et al., 2019). However, a large number of studies confirms that BNP is a protective factor of CAD and a self-regulator of the body’s pathological state. The release of BNP improves myocardial relaxation and response to the acute increase of ventricular volume by opposing sodium retention, vasoconstriction, and antidiuretic effects of the activated renin-angiotensin-aldosterone system (Daniels and Maisel, 2007). All of these findings suggest that BNP may also have a protective role in stroke.

As a result, available clinical observational studies investigating the association between NT-proBNP and risk of stroke show ambiguous results. The confounding factors of the observational studies may cause BNP levels to rise, but this increase is not one of the causes of stroke; and those studies cannot rule out some implicit risk factors of stroke.

**FIGURE 1:**
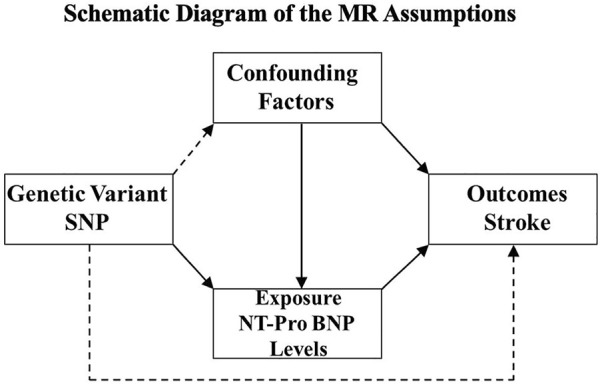
Schematic diagram of the MR assumptions. The arrows represent possible causal associations between variables. The dashed lines represent possible causal associations between variables that would violate the MR assumptions.

To circumvent the limitations of observational studies, Mendelian randomization (MR) analysis was used to improve causal inference. This technique is based on the premise that human genetic variants are randomly distributed among the population. This method may avoid the potential confounding factors within the exposure–outcome relationship and provide insight into the genetic association between the circulating NT-proBNP levels and ischemic stroke (Figure 1). Therefore, we conducted an MR analysis to investigate the causal effect of NT-proBNP on ischemic stroke and its subtypes (cardioembolism stroke, small-vessel occlusion stroke, and large-artery atherosclerosis stroke) by using three single-nucleotide polymorphisms (SNPs) (rs198389, rs13107325, rs11105306) associated with NT-pro-BNP level (Johansson et al., 2016).

## Manuscript Formatting

### Methods

#### Selection of Instrumental Variables

**FIGURE 2:**
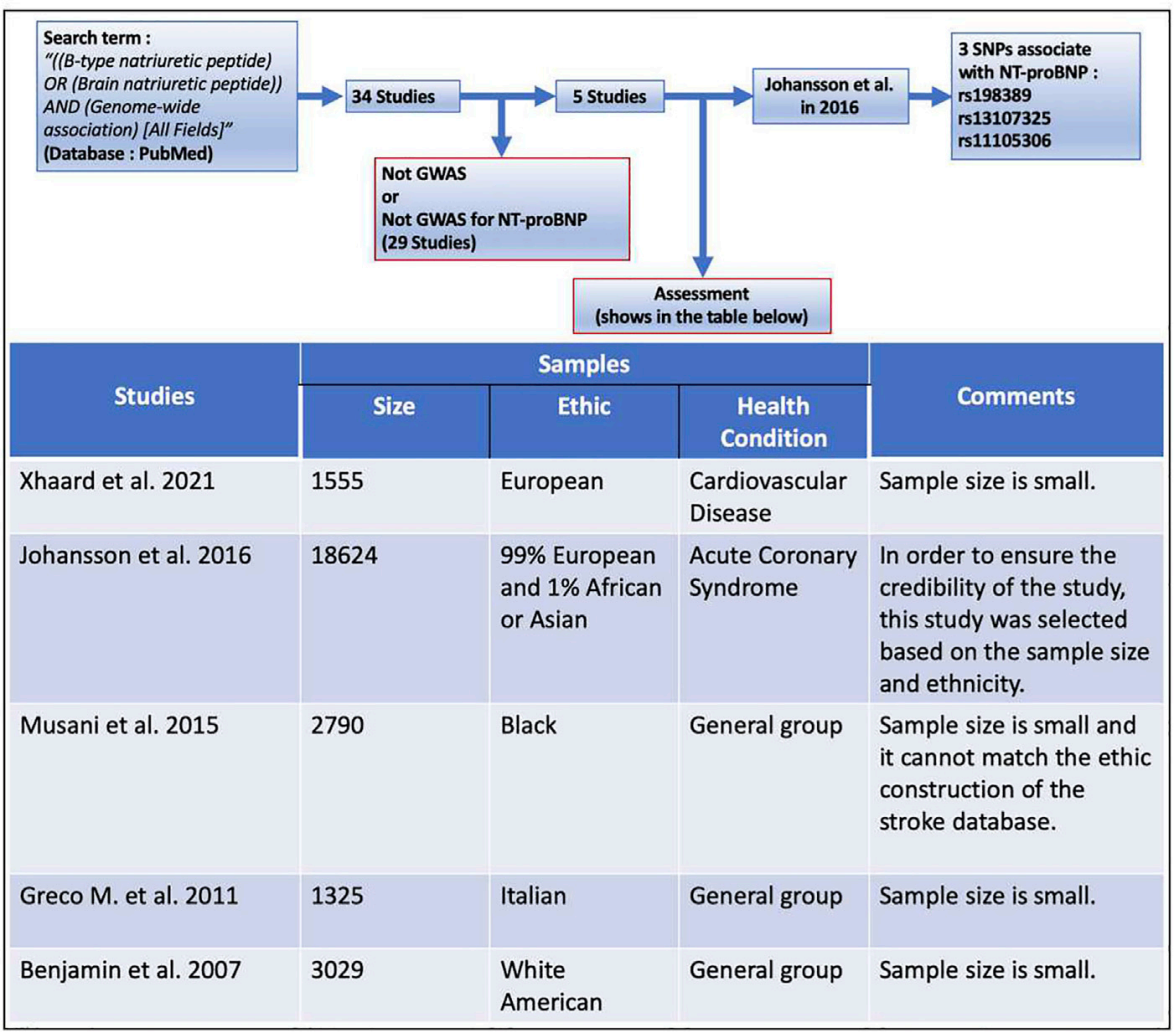
The retrieval process and inclusion/discharge criteria of instrumental variables.

To select SNPs associated with NT-proBNP as instrumental variables, the term “[(B-type natriuretic peptide) OR (Brain natriuretic peptide)] AND (Genome-wide association) (All Fields)” was searched in PubMed from 2005 to 2021, and the results showed a total of 34 articles (Supplementary Appendix). There are only five studies that found SNPs associated with NT-proBNP, of which the genome-wide association study (GWAS) performed by Johansson et al. (2016) was selected for our study (the retrieval process and inclusion/discharge criteria are shown in Figure 2). This GWAS of 18,624 individuals with acute coronary syndrome consisting of 99% European and 1% African or Asian identified two novel SNPs in SCL39A8 (rs13107325, pooled *p* = 5.99 × 10^−10^) and POC1B/GALANT4 (rs11105306, pooled *p* = 1.02 × 10^−16^) and confirmed one SNP (rs198389, pooled *p* = 1.07 × 10^−15^) that were all associated with the serum level of NT-proBNP. Among these three BNPs, rs198389 is proven to be associated with the level of NT-proBNP in several studies. The first study of this SNP was reported in 2007. This GWAS surrounding the natriuretic peptide precursor B (NPPB) gene with plasma BNP levels was performed in 2,970 adults from the general population (Takeishi et al., 2007). NPPB is on chromosome 1, encoding pre-proBNP. rs198389 is located in the NPPB promoter and has previously been found to influence promoter activity by interrupting an E-box consensus motif in the gene promoter (Meirhaeghe et al., 2007; Johansson et al., 2016). The rs13107325 is located in SLC39A8 on chromosome 4. It is a missense variant, which may cause an amino acid change at position 391 of the protein (Johansson et al., 2016). This substitution is predicted to be deleterious to the protein (Johansson et al., 2016). The rs11105306 is located in POC1B/GALANT4 on chromosome 12, which is in an intronic region with no obvious regulatory function (Johansson et al., 2016).

#### Outcome Data

The statistical data used for MR analysis of genetic associations with stroke was obtained from a multi-ancestry GWAS, including data from 521,612 individuals (67,162 cases and 454,450 controls) (Malik et al., 2018). These participants were selected from 29 investigations, consisting of ancestry groups from European (40,585 cases and 406,111 controls), East Asian (17,369 cases and 28,195 controls), African (5,541 cases and 15,154 controls), Latin American (865 cases and 692 controls), mixed Asian (365 cases and 333 controls), and South Asian (2,437 cases and 6,707 controls) (Malik et al., 2018). To avoid bias produced by a multi-ancestry population, we only used the data from the European group. The MEGASTROKE project was approved by relevant institutional review boards, and informed consent was obtained from each participant. The data set and basic information including sample size, age, and gender composition are presented in Table 1.

**TABLE 1:** The data set and basic information of the stroke GWAS in 2018.

Dataset	Stroke			Control		
	*N*	% Female	Mean AAO	*N*	% Female	Mean AAE
Metastroke	20,000	44.4%	67.1	19,326	49.9%	61.0
NINDS-SIGN	7,743	46.1%	66.5	17,970	—	—
Charge	4,348	67.0%	75.8	80,613	—	63.7
EPIC-CVD	4,347	48.0%	70.1	7,897	60.2%	64.1
Barcelona	520	41.9%	69.1	315	37.7%	67.5
Biobank Japan	16,256	36.8%	69.9	27,294	60.4%	57.5
CADISP	555	38.9%	43.7	9,259	—	—
Compass	5,541	—	—	15,154	—	—
Decode	5,520	44.2%	78.7	254,000	49.9%	53.3
Glasgow	599	49.7%	69.9	1,775	48.8%	69.6
Finland	501	40.9%	64.0	1,813	—	—
Hisayama	1,113	39.1%	69.7	901	40.5%	69.4
HVH—All	805	65.7%	68.3	1901	50.3%	66.4
Interstroke	2,429	44.3%	64.0	2,128	47.6%	62.5
MDC	202	34.7%	62.9	4,925	59.4%	57.2
RACE1	1,218	47.6%	50.1	1,158	47.0%	51.9
RACE2	1,167	—	—	4,035	—	—
SAHLSIS	298	40.9%	59.3	596	35.6%	56.8
SDS	52	46.2%	55.7	1,514	46.4%	53.0
SIFAP	981	38.9%	41.7	1825	50.7%	55.2
SLESS	546	42.1%	66.2	868	47.9%	58.7
UK young lacunar stroke DNA	1,403	32.8%	60.6	968	47.5%	59.7
ICH	1,545	45.1%	67.0	1,481	40.5%	65.3

AAO, age at onset; AAE, age at examination.

#### Statistical Analysis

First, we conducted genetic association analysis to evaluate the association between single NT-proBNP-associated SNPs and ischemic stroke and its three subtypes (cardioembolism, small-vessel occlusion, and large-artery atherosclerosis strokes). The significance threshold is *p* < .005, considering that many association studies for a single test changed the *p* value from .05 to .005, and the results with *p* values between .05 and .005 were considered to be suggestive of significance. Second, we conducted the MR analysis using three MR methods, including inverse-variance weighted (IVW), weighted median, and MR-Egger. IVW is the main MR analysis method, which combines the variant-specific Wald estimators by taking the inverse of their approximate variances as the corresponding weights (Bowden et al., 2016). Weighted median could derive consistent estimates when up to 50% of instruments are not valid (Bowden et al., 2016). MR-Egger could test the presence of potential pleiotropy and account for this potential pleiotropy using the MR-Egger intercept test (Burgess and Thompson, 2017). The odds ratio (OR) as well as 95% confidence interval (CI) of stroke corresponds to about 1 standard deviation (SD) in NT-proBNP level. All the statistical tests were completed using R Packages “Mendelian Randomization” (Yavorska and Burgess, 2017) and a *p* < .0042 (0.05/12 adjusted with Bonferroni method) was considered statistically significant; *p* between .05 and .0042 were considered suggestive of significance.

### Results and Discussion

**FIGURE 3:**
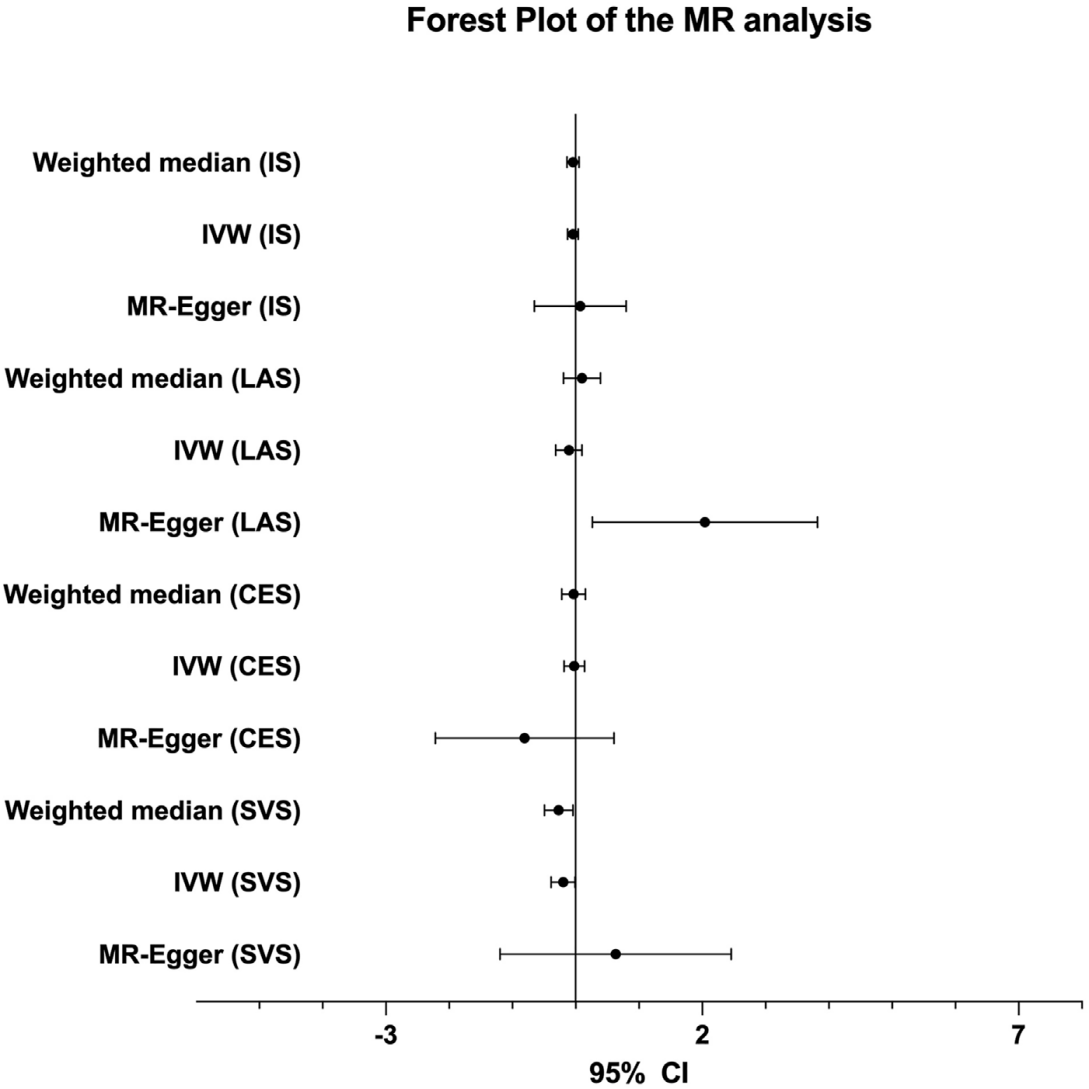
The forest plot of the MR analysis.

The genetic association analysis evaluating the association between single NT-proBNP-associated SNPs and ischemic stroke and its three subtypes shows that neither of those SNPs have significant association with ischemic stroke and subtypes, whereas only rs198389 has a suggestive association with LAS (95% CI 0.017∼0.116, *p* = .008686, .05 > *p* > .005) (Table 2). The MR analysis using three MR methods (IVW, weighted median, MR-Egger) shows no significant causal association between BNP levels and the risk of ischemic stroke. However, the weighted median and the IVW present suggestive association in small-vessel occlusion stroke (SVS) (weighted median: OR = −0.268, 95% CI −0.492∼−0.044, *p* = .019; IVW: OR = −0.199, 95% CI −0.389∼−0.009, *p* = .040) with no horizontal pleiotropy, which was identified with the MR-egger method (*p* = .499) (Table 3; Figure 3). In conclusion, the genetic association analysis shows that rs198389 has a suggestive association with LAS, and the MR analysis shows that NT-proBNP levels suggestively reduce the risk of SVS.

**TABLE 2:** The genetic association analysis of BNPs and ischemic stroke and its subtypes.

SNP	Stroke types	Allele1	Allele2	Freq1^a^	Effect	StdErr^b^	*p*-value
rs198389	AIS^c^	a	g	0.5846	0.0093	0.0103	0.367
	LAS^d^	a	g	0.5833	0.0667	0.0254	0.008686
	CES^e^	a	g	0.5851	−0.0126	0.0196	0.5212
	SVS^f^	a	g	0.5838	0.0406	0.0236	0.0856
rs13107325	AIS	t	c	0.0748	−0.0065	0.0215	0.7611
	LAS	t	c	0.0802	0.0206	0.0529	0.6965
	CES	t	c	0.0769	−0.0299	0.0435	0.4921
	SVS	t	c	0.0766	0.0234	0.0475	0.6219
rs11105306	AIS	t	c	0.2461	0.0058	0.0123	0.6384
	LAS	t	c	0.2432	−0.0213	0.0294	0.4695
	CES	t	c	0.2439	0.0146	0.0229	0.5232
	SVS	t	c	0.2447	0.0492	0.0271	0.06945

a Frequence.

b Standard error.

c Acute ischemic stroke.

d Large-artery atherosclerosis stroke.

e Cardioembolism stroke.

f Small-vessel occlusion stroke.

**TABLE 3:** MR analysis of association between 3 BNPs (rs198389, rs13107325, rs11105306) and ischemic stroke and its subtypes.

Stroke types	Method	Estimate	Std. error	95% CI	*p*-value
IS	Weighted median	−0.04	0.049	−0.136, 0.056	0.415
	IVW	−0.044	0.043	−0.129, 0.041	0.313
	MR-Egger	0.073	0.370	−0.653, 0.799	0.843
	MR-Egger (intercept)	−0.020	0.063	−0.144, 0.104	0.751
LAS	Weighted median	0.100	0.149	−0.191, 0.391	0.501
	IVW	−0.107	0.105	−0.314, 0.099	0.308
	MR-Egger	2.042	0.908	0.263, 3.821	0.024
	MR-Egger (intercept)	−0.371	0.156	−0.676, −0.066	0.017
CES	Weighted median	−0.031	0.095	−0.218, 0.156	0.746
	IVW	−0.023	0.083	−0.185, 0.139	0.779
	MR-Egger	−0.808	0.720	−2.219, 0.604	0.262
	MR-Egger (intercept)	0.135	0.123	−0.106, 0.376	0.273
SVS	Weighted median	−0.268	0.114	−0.492, −0.044	0.019
	IVW	-0.199	0.097	−0.389, −0.009	0.040
	MR-Egger	0.631	0.933	−1.197, 2.459	0.499
	MR-Egger (intercept)	−0.144	0.160	−0.458, 0.170	0.370

#### Analysis of the Negative Results

This MR study overcomes confounding risk factors and shows that there is no significant causal association between BNP levels and the risk of ischemic stroke, which is contrary to the results of most previous prospective studies. The discrepancy therein may be ascribed to the negligence of some hidden risk factors for stroke, which may cause BNP levels to rise without any causal association with stroke. In 2013, in a random community-based sample, Cannone et al. found that rs5065 was associated with increased cardiovascular risk by analyzing the phenotype associated with atrial natriuretic peptide (ANP) genetic variant rs5065. The rs5065 is a genetic variant and its minor allele encodes for an ANP with two additional arginines at the C-terminus, ANP-RR. This research also found that the endothelial hyperpermeability induced by chronic exposure to ANP-RR may predispose the subject to atherosclerotic disease. Interestingly, the minor allele of rs5065 is associated with higher BNP plasma values. The researchers hypothesized that higher levels of BNP might be originated from the deleterious effects caused by ANP-RR on the heart although it did not reveal any other CAD signs (Cannone et al., 2013). The rs5065 causes both ANP-RR and BNP levels to increase, but only ANP-RR is the causal factor. Pathways such as this may exist in the incidence of stroke and lead to controversial results. Although some evidence suggests causal relationship between natriuretic peptides and endothelial permeability, which might predispose to atherosclerosis and hemorrhages, some research shows that BNP may also have anti-inflammatory endothelial actions (Kuhn, 2012). These two actions are contrary to each other, which may explain the difference between the results mentioned above and our result.

#### Possible Explanations of the Suggestive Associations

The contradiction stated in the previous paragraph leads us to focus on the suggestive associations found in this study. The genetic association analysis shows that rs198389 alone has a suggestive association with LAS. The MR analysis shows that NT-proBNP levels have a suggestive positive causal effect on LAS in MR-Egger analysis (OR = 2.042, 95% CI 0.263–3.821, *p* = .024), but the MR-Egger intercept (95%CI −0.676∼−0.066, *p* = .017) is significantly different from zero, showing a pleiotropic effect on this outcome. The origin of loci may affect the results. In this study, the rs198389 locus came from a large population without special classification, and the other two loci came from people with ACS in GWAS performed by Johansson et al. In our study, genes as instrumental variables need to be absolutely associated with exposure factors. However, the association between NT-proBNP and the loci found in ACS patients is questionable. Therefore, we cannot conclude that NT-proBNP has no causal relationship with stroke merely based on this study. We chose the GWAS performed by Johansson et al. as the SNPs source because it has the largest sample size among all of the available GWAS of NT-proBNP (Figure 2). This is based on the idea that many of the current limitations of GWAS can be overcome to some extent by increasing sample sizes, which makes GWAS with larger sample sizes more reliable (Tam et al., 2019). Therefore, GWAS of NT-proBNP in general populations with a large sample size is anticipated to explore the relationship between SNP and stroke more accurately.

Interestingly, in our study, it is also implied that the serum level of NT-proBNP suggestively reduces the risk of SVS. The role of BNP in lowering blood pressure may be involved in the mechanism behind this phenomenon. BNP is released from the heart muscle in response to blood pressure and volume overload. Its main effects are reducing the preload of the heart by promoting diuresis and capillary permeability, which results in the reduction of the blood pressure (Goetze et al., 2020). In 2013, Wang et al. performed a retrospective study on the association between hypertension and different ischemic stroke subtypes, which involved 11,560 patients with ischemic stroke. The results show that hypertension is significantly related to recurrent stroke in patients with SVS, but not other subtypes of ischemic stroke (Wang et al., 2013). Taken together, we conclude that BNP can reduce the risk of SVS by lowering blood pressure. Whether BNP can reduce the risk of SVS needs to be verified by more accurate and credible studies in the future, which will help us form a better understanding of the pathogenesis and treatment of SVS.

#### Strengths and Limitations

Our MR study has several strengths. First, stroke is a complex disease with a large number of risk factors and pathophysiological pathways. However, in this study, the relationship between NT-proBNP and stroke was studied at the gene level with a large sample size and directly from the gene, which reduces the possibility of interference from implied risk factors. Second, in this study, the potential confounding factors caused by linkage disequilibrium may be reduced by using three independent genetic variants as instrumental variables. Third, we selected three MR methods to enhance the robustness of estimates. Fourth, three-stage pleiotropy analysis were performed, which may decrease the risk of pleiotropy.

Some limitations still exist in this MR analysis. First, the additional confounders cannot be completely ruled out as well as for the pleiotropy present in any MR study. Second, the obtained analysis results may be influenced by the population stratification, which cannot be fully ruled out. Third, the genetic relationship between NT-proBNP levels and stroke risk may be different in diverse genetic ancestries or ethnicities.

This genetic association should be further evaluated in other ancestries. Fourth, a replication study should be performed to ensure the accuracy and rigor of our original study. However, the GWAS of stroke we used as outcome data had very large sample size. It conducted meta-analyses of 29 studies, which involved every large size of stroke-related database before 2018. As we know, there are no other relative studies that have approximately the same order of magnitude as the previous GWAS. Replication studies should be performed with another large GWAS of ischemic stroke.

## Conclusion

This research provides evidence that there is no causal relationship between elevated NT-proBNP level and the risk of stroke. It is ineffective to use NT-proBNP as the target for stroke treatment and prevention. NT-proBNP plays an important role in ischemic stroke, but its function is not completely clear, and its association with stroke needs to be further explored.

## Data Availability

The original contributions presented in the study are included in the article/Supplementary Material, further inquiries can be directed to the corresponding author.

## Abbreviations

AAE, Age at examination; AAO, Age at onset; ACS, Acute coronary syndromes; AIS, Acute ischemic stroke; ANP, Atrial natriuretic peptide; ARIC, Atherosclerosis Risk in Communities; BNP, Brain natriuretic peptide; CAD, Coronary artery disease; CES, Cardioembolism stroke; CI, Confidence interval; GWAS, Genome-wide association study; IVW, Inverse-variance weighted; LAS, Large-artery atherosclerosis stroke; MR, Mendelian randomization; NPPB, Natriuretic peptide precursor B; NT-proBNP, N-terminal pro-brain natriuretic peptide; OR, Odds ratio; SD, Standard deviation; SNP, Single-nucleotide polymorphism; SVS, Small-vessel occlusion stroke.
