# Supplementary material for: Circulating N-Terminal Probrain Natriuretic Peptide Levels in Relation to Ischemic Stroke and Its Subtypes: A Mendelian Randomization Study
Source: Front Genet. 2022 Feb 22;13:795479. doi: 10.3389/fgene.2022.795479 (PMC8902306; doi:10.3389/fgene.2022.795479)
Supplement: Supplementary file 1 [file DataSheet1.docx]

# Appendix

The initial searching result in PubMed :

1. Pausova Z, Gaudet D, Gossard F, Bernard M, Kaldunski ML, Jomphe M, et al. Genome-wide scan for linkage to obesity-associated hypertension in French Canadians. *Hypertension* (2005) 46(6):1280-5. Epub 2005/10/12. doi: 10.1161/01.HYP.0000188049.23233.fb. PubMed PMID: 16216983.

2. Benjamin EJ, Dupuis J, Larson MG, Lunetta KL, Booth SL, Govindaraju DR, et al. Genome-wide association with select biomarker traits in the Framingham Heart Study. *BMC Med Genet* (2007) 8 Suppl 1:S11. Epub 2007/10/16. doi: 10.1186/1471-2350-8-S1-S11. PubMed PMID: 17903293; PubMed Central PMCID: PMCPMC1995615.

3. Choquet H, Cavalcanti-Proenca C, Lecoeur C, Dina C, Cauchi S, Vaxillaire M, et al. The T-381C SNP in BNP gene may be modestly associated with type 2 diabetes: an updated meta-analysis in 49 279 subjects. *Hum Mol Genet* (2009) 18(13):2495-501. Epub 2009/04/21. doi: 10.1093/hmg/ddp169. PubMed PMID: 19377085.

4. Delfino KR, Southey BR, Sweedler JV, Rodriguez-Zas SL. Genome-wide census and expression profiling of chicken neuropeptide and prohormone convertase genes. *Neuropeptides* (2010) 44(1):31-44. Epub 2009/12/17. doi: 10.1016/j.npep.2009.11.002. PubMed PMID: 20006904; PubMed Central PMCID: PMCPMC2814002.

5. Hiura Y, Tabara Y, Kokubo Y, Okamura T, Miki T, Tomoike H, et al. A genome-wide association study of hypertension-related phenotypes in a Japanese population. *Circ J* (2010) 74(11):2353-9. Epub 2010/09/30. doi: 10.1253/circj.cj-10-0353. PubMed PMID: 20877124.

6. Del Greco MF, Pattaro C, Luchner A, Pichler I, Winkler T, Hicks AA, et al. Genome-wide association analysis and fine mapping of NT-proBNP level provide novel insight into the role of the MTHFR-CLCN6-NPPA-NPPB gene cluster. *Hum Mol Genet* (2011) 20(8):1660-71. Epub 2011/01/29. doi: 10.1093/hmg/ddr035. PubMed PMID: 21273288; PubMed Central PMCID: PMCPMC3063986.

7. Piran S, Liu P, Morales A, Hershberger RE. Where genome meets phenome: rationale for integrating genetic and protein biomarkers in the diagnosis and management of dilated cardiomyopathy and heart failure. *J Am Coll Cardiol* (2012) 60(4):283-9. Epub 2012/07/21. doi: 10.1016/j.jacc.2012.05.005. PubMed PMID: 22813604.

8. Dadu RT, Dodge R, Nambi V, Virani SS, Hoogeveen RC, Smith NL, et al. Ceruloplasmin and heart failure in the Atherosclerosis Risk in Communities study. *Circ Heart Fail* (2013) 6(5):936-43. Epub 2013/07/19. doi: 10.1161/CIRCHEARTFAILURE.113.000270. PubMed PMID: 23861484; PubMed Central PMCID: PMCPMC3908901.

9. Rubattu S, Giusti B, Lotta LA, Peyvandi F, Cotugno M, Stanzione R, et al. Association of a single nucleotide polymorphism of the NPR3 gene promoter with early onset ischemic stroke in an Italian cohort. *Eur J Intern Med* (2013) 24(1):80-2. Epub 2012/09/22. doi: 10.1016/j.ejim.2012.09.002. PubMed PMID: 22995222.

10. Tabara Y, Igase M, Okada Y, Nagai T, Uetani E, Kido T, et al. Association of Chr17q25 with cerebral white matter hyperintensities and cognitive impairment: the J-SHIPP study. *Eur J Neurol* (2013) 20(5):860-2. Epub 2012/10/02. doi: 10.1111/j.1468-1331.2012.03879.x. PubMed PMID: 23020117.

11. Vogel B, Keller A, Frese KS, Leidinger P, Sedaghat-Hamedani F, Kayvanpour E, et al. Multivariate miRNA signatures as biomarkers for non-ischaemic systolic heart failure. *Eur Heart J* (2013) 34(36):2812-22. Epub 2013/07/19. doi: 10.1093/eurheartj/eht256. PubMed PMID: 23864135.

12. Beygui F, Wild PS, Zeller T, Germain M, Castagne R, Lackner KJ, et al. Adrenomedullin and arterial stiffness: integrative approach combining monocyte ADM expression, plasma MR-Pro-ADM, and genome-wide association study. *Circ Cardiovasc Genet* (2014) 7(5):634-41. Epub 2014/07/24. doi: 10.1161/CIRCGENETICS.113.000456. PubMed PMID: 25053723; PubMed Central PMCID: PMCPMC5865635.

13. Jorde A, Bach P, Witt SH, Becker K, Reinhard I, Vollstadt-Klein S, et al. Genetic variation in the atrial natriuretic peptide transcription factor GATA4 modulates amygdala responsiveness in alcohol dependence. *Biol Psychiatry* (2014) 75(10):790-7. Epub 2013/12/10. doi: 10.1016/j.biopsych.2013.10.020. PubMed PMID: 24314346.

14. Abbasi A. Mendelian randomization studies of biomarkers and type 2 diabetes. *Endocr Connect* (2015) 4(4):249-60. Epub 2015/10/09. doi: 10.1530/EC-15-0087. PubMed PMID: 26446360; PubMed Central PMCID: PMCPMC4654400.

15. Cakmak HA, Coskunpinar E, Ikitimur B, Barman HA, Karadag B, Tiryakioglu NO, et al. The prognostic value of circulating microRNAs in heart failure: preliminary results from a genome-wide expression study. *J Cardiovasc Med (Hagerstown)* (2015) 16(6):431-7. Epub 2015/02/03. doi: 10.2459/JCM.0000000000000233. PubMed PMID: 25643195.

16. Kato N, Loh M, Takeuchi F, Verweij N, Wang X, Zhang W, et al. Trans-ancestry genome-wide association study identifies 12 genetic loci influencing blood pressure and implicates a role for DNA methylation. *Nat Genet* (2015) 47(11):1282-93. Epub 2015/09/22. doi: 10.1038/ng.3405. PubMed PMID: 26390057; PubMed Central PMCID: PMCPMC4719169.

17. Lieb W, Chen MH, Teumer A, de Boer RA, Lin H, Fox ER, et al. Genome-wide meta-analyses of plasma renin activity and concentration reveal association with the kininogen 1 and prekallikrein genes. *Circ Cardiovasc Genet* (2015) 8(1):131-40. Epub 2014/12/06. doi: 10.1161/CIRCGENETICS.114.000613. PubMed PMID: 25477429; PubMed Central PMCID: PMCPMC4354880.

18. Musani SK, Fox ER, Kraja A, Bidulescu A, Lieb W, Lin H, et al. Genome-wide association analysis of plasma B-type natriuretic peptide in blacks: the Jackson Heart Study. *Circ Cardiovasc Genet* (2015) 8(1):122-30. Epub 2015/01/07. doi: 10.1161/CIRCGENETICS.114.000900. PubMed PMID: 25561047; PubMed Central PMCID: PMCPMC4426827.

19. Ooi JY, Tuano NK, Rafehi H, Gao XM, Ziemann M, Du XJ, et al. HDAC inhibition attenuates cardiac hypertrophy by acetylation and deacetylation of target genes. *Epigenetics* (2015) 10(5):418-30. Epub 2015/05/06. doi: 10.1080/15592294.2015.1024406. PubMed PMID: 25941940; PubMed Central PMCID: PMCPMC4622459.

20. Padmanabhan S, Caulfield M, Dominiczak AF. Genetic and molecular aspects of hypertension. *Circ Res* (2015) 116(6):937-59. Epub 2015/03/15. doi: 10.1161/CIRCRESAHA.116.303647. PubMed PMID: 25767282.

21. Johansson A, Eriksson N, Lindholm D, Varenhorst C, James S, Syvanen AC, et al. Genome-wide association and Mendelian randomization study of NT-proBNP in patients with acute coronary syndrome. *Hum Mol Genet* (2016) 25(7):1447-56. Epub 2016/02/26. doi: 10.1093/hmg/ddw012. PubMed PMID: 26908625.

22. Solomon T, Smith EN, Matsui H, Braekkan SK, Consortium I, Wilsgaard T, et al. Associations Between Common and Rare Exonic Genetic Variants and Serum Levels of 20 Cardiovascular-Related Proteins: The Tromso Study. *Circ Cardiovasc Genet* (2016) 9(4):375-83. Epub 2016/06/23. doi: 10.1161/CIRCGENETICS.115.001327. PubMed PMID: 27329291; PubMed Central PMCID: PMCPMC4982757.

23. Turer AT, Scherer PE. Adiponectin: Just Along for the Ride? *Circ Res* (2016) 119(3):407-8. Epub 2016/07/28. doi: 10.1161/CIRCRESAHA.116.309226. PubMed PMID: 27458194; PubMed Central PMCID: PMCPMC4966901.

24. Wang JJ, Rau C, Avetisyan R, Ren S, Romay MC, Stolin G, et al. Genetic Dissection of Cardiac Remodeling in an Isoproterenol-Induced Heart Failure Mouse Model. *PLoS Genet* (2016) 12(7):e1006038. Epub 2016/07/08. doi: 10.1371/journal.pgen.1006038. PubMed PMID: 27385019; PubMed Central PMCID: PMCPMC4934852.

25. Armando I. News From the Heart Natriuretic System. *Circ Cardiovasc Genet* (2017) 10(6). Epub 2017/12/15. doi: 10.1161/CIRCGENETICS.117.002011. PubMed PMID: 29237682; PubMed Central PMCID: PMCPMC7041670.

26. Morrison AC, Huang Z, Yu B, Metcalf G, Liu X, Ballantyne C, et al. Practical Approaches for Whole-Genome Sequence Analysis of Heart- and Blood-Related Traits. *Am J Hum Genet* (2017) 100(2):205-15. Epub 2017/01/17. doi: 10.1016/j.ajhg.2016.12.009. PubMed PMID: 28089252; PubMed Central PMCID: PMCPMC5294677.

27. Salo PP, Havulinna AS, Tukiainen T, Raitakari O, Lehtimaki T, Kahonen M, et al. Genome-Wide Association Study Implicates Atrial Natriuretic Peptide Rather Than B-Type Natriuretic Peptide in the Regulation of Blood Pressure in the General Population. *Circ Cardiovasc Genet* (2017) 10(6). Epub 2017/12/15. doi: 10.1161/CIRCGENETICS.117.001713. PubMed PMID: 29237677; PubMed Central PMCID: PMCPMC6072381.

28. Costas J. The highly pleiotropic gene SLC39A8 as an opportunity to gain insight into the molecular pathogenesis of schizophrenia. *Am J Med Genet B Neuropsychiatr Genet* (2018) 177(2):274-83. Epub 2017/05/31. doi: 10.1002/ajmg.b.32545. PubMed PMID: 28557351.

29. Ko D, Benson MD, Ngo D, Yang Q, Larson MG, Wang TJ, et al. Proteomics Profiling and Risk of New-Onset Atrial Fibrillation: Framingham Heart Study. *J Am Heart Assoc* (2019) 8(6):e010976. Epub 2019/03/08. doi: 10.1161/JAHA.118.010976. PubMed PMID: 30841775; PubMed Central PMCID: PMCPMC6475036.

30. Ananthan K, Lyon AR. The Role of Biomarkers in Cardio-Oncology. *J Cardiovasc Transl Res* (2020) 13(3):431-50. Epub 2020/07/10. doi: 10.1007/s12265-020-10042-3. PubMed PMID: 32642841; PubMed Central PMCID: PMCPMC7360533.

31. Raffield LM, Lu AT, Szeto MD, Little A, Grinde KE, Shaw J, et al. Coagulation factor VIII: Relationship to cardiovascular disease risk and whole genome sequence and epigenome-wide analysis in African Americans. *J Thromb Haemost* (2020) 18(6):1335-47. Epub 2020/01/28. doi: 10.1111/jth.14741. PubMed PMID: 31985870; PubMed Central PMCID: PMCPMC7274883.

32. Theriault S, Sjaarda J, Chong M, Hess S, Gerstein H, Pare G. Identification of Circulating Proteins Associated With Blood Pressure Using Mendelian Randomization. *Circ Genom Precis Med* (2020) 13(1):e002605. Epub 2020/01/14. doi: 10.1161/CIRCGEN.119.002605. PubMed PMID: 31928076.

33. Tian D, Zhang L, Zhuang Z, Huang T, Fan D. A Mendelian randomization analysis of the relationship between cardioembolic risk factors and ischemic stroke. *Sci Rep* (2021) 11(1):14583. Epub 2021/07/18. doi: 10.1038/s41598-021-93979-y. PubMed PMID: 34272412; PubMed Central PMCID: PMCPMC8285403.

34. Xhaard C, Rouget R, Vodovar N, Le Floch E, Dandine-Roulland C, Wagner S, et al. Impact of natriuretic peptide polymorphisms on diastolic and metabolic function in a populational cohort: insights from the STANISLAS cohort. ESC Heart Fail (2021). Epub 2021/11/05. doi: 10.1002/ehf2.13674. PubMed PMID: 34734498.
